# Statistical considerations for stopping systemic lupus erythematosus clinical trials earlier

**DOI:** 10.1186/s13075-015-0874-0

**Published:** 2015-12-04

**Authors:** Robert A. Lew, Matthew H. Liang, Gheorghe Doros

**Affiliations:** Department of Biostatistics, Boston University School of Public Health, Boston, MA 02118 USA; VA Cooperative Studies Program, VA Boston Healthcare System, Boston, MA 02115 USA; Section of Clinical Sciences, Division of Rheumatology, Immunology, and Allergy, Brigham and Women’s Hospital, Boston, MA 02115 USA; Section of Rheumatology, VA Boston Healthcare System, Boston, MA 02115 USA

## Abstract

Group sequential designs are used to potentially shorten randomized clinical trials and thereby reduce subject burden, improve safety, and save time and resources. Clinical trials comparing treatments for systemic lupus erythematosus (SLE) might adopt such designs if the ordinal outcome scales for SLE, such as the Systemic Lupus Activity Measure and Systemic Lupus Erythematosus Disease Activity Index, were more like continuous outcome scales with interval properties. After describing the basic features of sequential trials and highlighting some major issues in their design, we propose approaches that mitigate these issues. In particular, high-speed computing has accelerated advances in sequential design, making available a variety of designs that can be implemented with minimal technical support. The challenge now is to understand the concepts behind such flexible designs and then to apply them to improve studies of SLE.

## Introduction

Terminating a clinical trial as soon as a robust result becomes evident is an ethical and practical imperative and minimizes exposure of volunteer participants to potentially ineffective or toxic treatment. Group sequential clinical trial designs are a means to this end. This paper discusses and outlines the process and the methods of sequential designs in systemic lupus erythematosus (SLE), a disease like no other in its protean and variable manifestations. To the best of our knowledge no one has implemented such a design for an SLE study. One reason may be that many SLE outcome measures have ordinal rather than interval properties. Ideally, a sequential trial should have an a priori definition of clinically meaningful change on an interval scale. We propose methods to transform an ordinal measure into a measure closer to this ideal. Studies in the systemic rheumatic conditions have employed composite outcome scales to capture the full impact of these illnesses on the individual. These combine levels of disability, symptoms, and physiological biomarkers. Such disparate elements do not simply add up, but are combined into an ordinal scale with or without weights. The weighting may be done implicitly or inferred indirectly by expert clinicians. Compared with continuous outcome measures, ordinal measures such as the British Isles Lupus Assessment Group (BILAG), Systemic Lupus Activity Measure (SLAM) and Systemic Lupus Erythematosus Disease Activity Index (SLEDAI) [1] are not optimal for tracking the progression of disease over time. The clinical importance of a change of one unit in an ordinal scale, from n to n + 1, may vary depending on the value of n. Ideally, if comparing mean treatment effects with Student’s *t*-test, the difference between means should have an unambiguous clinical interpretation, regardless of the particular mean values.

Continuous measures, however, also fall short. For example, a decrease in systolic blood pressure from 240 to 200 mm Hg has a different meaning and clinical significance than a decrease from 140 to 100 mm Hg. Therefore, both the absolute and the relative changes are needed to interpret a 40 mm Hg blood pressure drop.

This paper addresses this problem in the context of a sequential randomized clinical trial. Simple ‘one-stop’ trials have a fixed study period, such as 1 year, when they stop and test the null hypothesis that treatment effects are equal. Typical sequential trials plan on testing the null hypothesis several times during the study period; for example, a 1-year study might test at 3, 6, 9, and finally 12 months. At each time-point, an interim analysis is done to decide whether to stop or to continue the trial. The study may stop early either because the experimental treatment appears effective (and highly statistically significant) or because it appears ineffective and futile (with virtually no chance of reaching statistical significance by the end of the study).

## Interim analysis

**Fig. 1:**
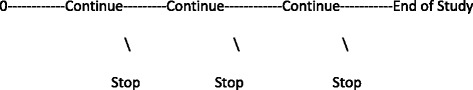
The pattern of decisions in a sequential trial

Sequential analyses periodically test a null hypothesis while the data accrue. Each interim test either stops or continues the study. The theory of sequential analysis largely originates with the work of Abraham Wald [2]. Driven out of Austria by the Nazis, his theoretical work became the basis of mathematical statistics [3] and his applied work led to major advances in manufacturing quality control, insurance, and sequential analysis. His work on the problem of WW2 bomber losses to enemy fire [4] led to better strategies that reduced losses. In medical research he showed how early stopping in a clinical trial could preserve resources with just a few more subjects than needed in a one-stop trial. Many advances in the design of sequential trials followed and then accelerated once high-speed computing became widely available. Chapter 1 of the Jennison and Turnbull seminal text *Group Sequential Trials* traces the history [5]. This text describes most of the methods currently used by the pharmaceutical industry and in academic, medical, and government organizations. Many designs have become feasible because only with high-speed computer simulation can one calculate power and type I error. Figure 1 indicates why. Each opportunity to stop the trial depends on all of the preceding decisions to continue to accrue data. The corresponding equations seldom have simple solutions.

## Alpha spending

A simple ‘one-stop’ design performs only one test when the trial ends, usually with 90 % power with a type I error of 5 %. Type I error is also called ‘alpha level’ or simply ‘alpha’. Sequential trials make several tests. We cannot use alpha = 5 % (a type I error of 5 %) at every interim analysis [6]. If we do so, as in Fig. 1, the actual type I error is about 20 % = 4 × 5 %, far too large a chance to mistakenly reject the null hypothesis. One should regard type I error of 5 % as if it were alpha = $5 in a bank account. In Fig. 1, you might spend $1 of alpha at each interim analysis and then spend $2 of alpha at the end, so-called ‘alpha spending’ [7]. Studies with interim analyses must distribute the type I error over all the potential stopping times. The final test must have type I error <5 % because some type I error was spent earlier. Simulation allows one to explore a wide range of spending plans to find an ‘optimal’ plan. No plan is actually optimal because all choices involve tradeoffs between minimal sample size and maximal power.

## Group sequential trials

Most clinical trials in SLE slowly accrue fewer than 10 patients from multiple sites during a year. For example, consider a 100-day SLE clinical trial that enrolls one patient per day. As in Fig. 1, interim analyses might occur at 25, 50, and 75 days. If the treatment result is immediate, then at 25 days we would analyze 25 results, at 50 days 50 results, and so on. The results accrue in groups of 25, hence the term ‘group sequential trials’.

Slow accrual of evaluable participants or those who reach a pre-specified endpoint adds complexity. Firstly, to avoid a hasty decision when the sample size is small, many designs make it very hard to reject the null hypothesis at the first interim analysis and gradually make it easier to reject it at the later interim analyses. Secondly, treatment outcomes in SLE are seldom immediate, so that, in the example above, only some of the 25 enrolled may be evaluable on day 25, only some of the 50 enrolled evaluable on day 50, and so on. Thirdly, survival (time-to-event) analyses have to account for the varying amounts of follow-up time. Substantial computer simulations can search for an ‘optimal’ design that addresses all these issues, but experienced clinicians must play a major role to ensure the optimality criteria are practical and clinically realistic [8].

## The O’Brien-Fleming design

Many sequential designs begin by assuming the test statistic, such as the difference between means, has a normal distribution. If the two treatments are labeled ‘A’ and ‘B’ , then at each interim analysis we would compare the mean of A, ā, to the mean of B, $\overline{\mathrm{b}}$. The null hypothesis, H0, is that the means do not differ, a zero difference. As patients accrue, the standard error of each sample mean tends to decrease. At each time let the difference be $\mathrm{d}=\overline{\mathrm{b}}-\overline{\mathrm{a}}$. Set z = d/sterr(d), where z is normally distributed with standard deviation 1 and sterr(d) is the standard error of d. Thus, as in Fig. 1, for three interim tests and one final test, if we did not stop early, during the study we would have observed four differences and their corresponding four observed z-scores, z_1_, z_2_, z_3_, and z_4_.

The hypothesis tests compare the observed z-scores to pre-specified cutoff Z-values. For a one-stop test of hypothesis with type I error of 5 % under the normal distribution the typical cutoff Z-value for a significant result is 1.96, for which the probability P(−1.96 < *z* < 1.96) = 0.95. Test statistics with values of z between the cutoff values, −1.96 and 1.96, are not significant and those with values outside this interval are significant.

Because of alpha-spending, all four z-cutoff values for a sequential test must exceed 1.96. An overly safe set of cutoff Z-values is 2.57, 2.57, 2.57, and 2.32 because P(|z| ≥ 2.57) = 0.01, P(|z| ≥ 2.32) = 0.02, and the sum of the four values of alpha would be 0.01 + 0.01 + 0.01 + 0.02 = 0.05. This ignores the fact that because the data used to calculate each successive test statistic contain all the previous data, the tests are positively correlated.

The O’Brien-Fleming rule starts with very a high cutoff Z-value and then declines over time [5]. For this example, the four cutoff Z-values are 4.048, 2.862, 2.337, and finally 2.024 [5]. By starting so high at 4.048, we spend very little alpha. Thus, we can finish at 2.024, a cutoff Z-value not much larger than 1.96. Ignoring the positive correlation, the corresponding sum of alpha values is 0.001 + 0.004 + 0.019 + 0.042 = 0.066. Fortunately, because the O’Brien-Fleming rule accounts for this correlation, the actual overall type I error is 5 %, even though the sum of the alpha values is 6.6 %. We pay for this with a small increase in total sample size; if a one-stop design needs 1000 subjects, then this sequential design needs 1024 subjects, a 2.4 % increase. Tables listing the cutoff Z-values and the increases in sample size appear in the Jennison and Turnbull text [5] along with explanatory material and examples. Also, one may obtain these values from PROC Seqdesign in the SAS statistical package (SAS version 9.3, SAS Institute Inc., Cary, NC, USA) and the program Clinfun in the R language online library of functions [9].

Applied to the design in Fig. 1, the O’Brien-Fleming test increases sample size, but provides three chances to stop early, but not for futility—that is, stopping early because the treatment difference is so small that gathering more data as planned has little or no chance to reject the null hypothesis [5]. More often than not, treatment differences are smaller than expected and seldom much larger than expected. Thus, in many studies an O’Brien-Fleming design with a very conservative option to stop for futility can shorten a study and save a lot of resources.

## Bayesian designs

High-speed computing allows us to explore many sets of cutoff Z-values to either reject the null hypothesis or declare futility. The Bayesian approach to design allows such a flexible approach, but adds terminology and intensive computation. Futility becomes easier to incorporate into the design [10, 11]. The logic of Bayesian inference for sequential designs resembles the logic of differential diagnosis and ‘trials of therapy’ when a physician works through a sequence of treatments with a patient until by trial and error they find the most effective treatment.

For Bayesian designs, however, physicians must specify prior opinions or beliefs about a meaningful difference between treatment effects, a challenging issue when using ordinal scales to score overall SLE manifestations or disease activity. To avoid bias it is critical to blind outcome assessment of subjectively rated phenomena. Therefore, Bayesian analysis requires ‘model criticism’ , an exploration of a wide range of prior assumptions to confirm or not confirm the results of the treatment comparison. These extra steps usually require guidance from a statistician and very complex computer simulation.

**Fig. 2:**
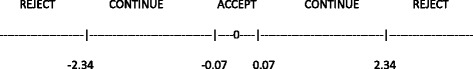
Cutoff Z-values for stopping to reject the null hypothesis (H0), stopping for futility, or continuing

Futility adds a second set of cutoff Z-values that are close to zero, indicative of a small difference between treatment means. In the Fig. 1 example, if the third interim analysis occurred at 9 months, we might reject the null hypothesis, H0, if the absolute value of the observed z-score is >2.34, accept H0 if <0.07 (a typical value for a futility cutoff), or continue. The 9-month cutoff Z-values partition the interval into five subintervals as in Fig. 2.

The term ‘ACCEPT’ means that it is futile to continue and more data are unlikely to lead us to reject H0. Conservative practice in clinical trials calls for two-sided tests; that is, reject if treatment A effects are significantly larger or smaller than treatment B effects. Thus, with a futility stopping option, the study continues unless the absolute treatment difference is either too large or too small.

## Ordinal scales

SLE is a multisystem disease with protean and varied manifestation and symptoms. As a consequence, measuring outcome has relied on multidimensional scales or composite indices for SLE, all of which yield ordinal data at best. Some scales are not even ordinal. The classic example, the ad hoc visual analog scale, asks a patient to mark a point on a 10-cm line to indicate, for example, their level of pain, with 0 for ‘no pain’ and 10 for ‘worst pain ever’ anchoring the ends of the line [12]. Each patient has a unique scale and their scales are logically incongruous; that is, patients who mark ‘5’ need not have the same level of pain. Similarly, the five-point Likert scale from ‘strongly agree’ to ‘strongly disagree’ is incongruous across people [13]. To make evaluation practical and for simplicity sake, we ignore such errors in measurement, although there are statistical methods that address this problem [14] (Table 1).

**Table 1:** Approximate extreme values of some ordinal outcome scales for systemic lupus erythematosus

Scale^a^	Minimum and maximum	Description
SLAM	0 to 84	Systemic Lupus Activity Measure
SLEDAI	0 to 108	Systemic Lupus Erythematosus Disease Activity Index
BILAG	0 to 180	British Isles Lupus Assessment Group
ECLAM	0 to 15.5	European Consensus Lupus Activity Measure

^a^Listing of items for each scale, and minimum and maximum scores obtained from Lam and Petri [1]

## Recalibrating an ordinal scale

We can simplify an ordinal scale to form a binary outcome. This was done, for example, in the Belimumab trial, where success was defined as a reduction of four or more in the Safety of Estrogens in Lupus Erythematosus-SLEDAI score [15]. This simple approach discards information, but the clinical importance of a reduction of four may vary depending on the baseline score.

Another instructive example comes from stroke studies in which the modified Rankin Scale is often used to evaluate patients 90 days after an incident of stroke [16] (Table 2).

**Table 2:** The seven categories of the modified Rankin score and an associated utility score

	Modified Rankin score value						
	0	1	2	3	4	5	6
Meaning of mRS value	No symptoms	No significant disability	Slight disability	Moderate disability	Moderately severe disability	Severe disability	Dead
Utility^a^	100	90	70	50	30	1	1

^a^These utilities are fabricated. mRS, modified Rankin score

Many studies reduce the modified Rankin Scale score to a binary outcome with success defined as a score of 2 or less, but others have used 1 or less [17]. Experts do not always agree on how to define success. One way to retain more detail is to assign clinically meaningful utilities to each value to allow comparison of mean treatment utilities as if the outcome measure were a continuous interval scale [18].

## Response criteria for systemic lupus erythematosus

The American College of Rheumatology (ACR) organized a working group in 2002 to develop standards for the evaluation of therapeutic interventions for patients with SLE [19]. It attempted to develop a data-driven consensus on meaningful clinical change over time ‘to help investigators develop sample size estimates based on meaningful effect sizes and to gauge the clinical relevance of any observed change in disease activity’. Experts on SLE used a secure web-based survey to review a sample of actual patient histories chosen from 310 carefully abstracted, longitudinal, and uniformly formatted SLE patient case histories, over a 2- or 6-month interval. The cases were assessed by several experts as to the degree of change—either ‘worse’ , ‘no change’ or ‘improved’ and blinded to the independently scored disease activity measures listed in Table 1. For example, if a change, Δ, in a scale was 4 units, then the aggregated data allowed estimation of the three probabilities (P) that add up to 1.0: P(Worse|(Δ = 4)) = 0.82, P(No change|(Δ = 4)) = 0.12, and P(Better|(Δ = 4)) = 0.06.

A consensus of the experts in this ACR working group interpreted a rise of 4 units for this particular scale as worsening disease. The study did not determine whether or not rises from different baseline values, such as 0 to 4, 1 to 5, and 2 to 6, represented similar levels of clinical change , but suggested that it sufficed to consider only Δ = 4. Also, Fig. 2 of the ACR paper [19] supported a symmetric interpretation, that a decrease of 4 implied clinical improvement.

## Increasing the uniformity in levels of clinical change

A general approach is to recalibrate an ordinal scale to make changes of the same size more clinically uniform. The following illustrative example indicates how one might recalibrate SLAM scores that vary from 0 to 84, but could be applied to any ordinal scale (SLE or not). The interval width of 20 is arbitrary. First, ask a group of experts to construct intervals that map the raw scores into a few categories of increasing severity (Table 3). The underlying assumption is that the changes from negligible to mild, from mild to moderate, and from moderate to severe are roughly equal in terms of clinical importance. Next, recalibrate the raw scores so that each category is 20 units wide (Table 4).

**Table 3:** Raw systemic lupus activity measure scores divided into four categories

	Raw systemic lupus activity measure score			
	0 to 20	21 to 40	41 to 50	51 to 84
Disease severity	1	2	3	4
Description	Negligible	Mild	Moderate	Severe

**Table 4:** Systemic lupus activity measure score categories recalibrated to have equal width

Raw SLAM categories	0 to 20	21 to 40	41 to 50	51 to 84
Uniform width categories	0 to 20	21 to 40	41 to 60	61 to 80

In Table 4, the raw scores from 41 to 50 then stretch into scores from 41 to 60 while raw scores from 51 to 84 squeeze into scores from 61 to 80. Hence, we stretch and squeeze the raw scale to give differences between values a more similar clinical meaning. Then the difference between mean uniform-width SLAM scores should have a more clinically consistent meaning than the difference between mean raw scores. While simple to describe, such a process requires a consensus among experts. The example above outlines the process, but a genuine effort by experts would require a major effort. Ideally, the experts would make uniform-width intervals in several distinct ways to check that a significant statistical result was not merely an artifact of the process. For example, the range of scores could be divided into six categories.

## A hypothetical systemic lupus erythematosus example

**Fig. 3:**
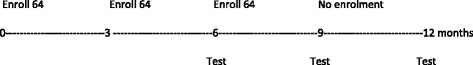
An example of the pattern of enrolment in a group sequential trial

A 12-month study compares two SLE treatments, A and B, using the smoothed SLAM score as the outcome measure. The study enrolls a total of 192 subjects, 96 per study arm. Each patient is treated for 3 months and the 3-month SLAM score is the primary outcome. Beginning at time 0, during the first 3 months 64 patients are enrolled, 32 receive A and 32 receive B. From the beginning of month 3 to the end of month 5 and then from the beginning of month 6 to the end of month 8 exactly the same enrolment occurs. During the last 3 months no subjects enroll. By the end of the year the last patient enrolled will have completed treatment. Figure 3 illustrates this enrolment pattern. For simplicity, we assume no drop outs.

The interim analysis tests are right-shifted along the time axis. The test at the beginning of month 6 can only compare the outcomes of the first 64 patients enrolled during the first 3 months, the last of whom completed 3 months' of follow-up at the end of month 5. The test at 9 months evaluates 128 subjects and the test at 12 months evaluates 192 subjects.

To add a realistic concern, suppose the experts undertook the study hoping that a new treatment A would prove superior to a standard treatment B. In terms of SLAM scores, a lower score is superior. Then, if during the study the results went in the wrong direction (subjects on treatment B had lower scores) and the observed mean difference, $d=\overline{b}-\overline{a}$ < 0, we might stop the trial for futility. Typically, we use a conservative two-sided null hypothesis (H0) and a two-sided alternative hypothesis (HA). Assuming differences go in the direction hoped for by the experts, then with type I error = 5 % and power = 90 % under an O’Brien-Fleming design, the three cutoff Z-values to reject H0 would be 3.47, 2.45, and 2.00, with corresponding type I errors of 0.0005, 0.014, and 0.045. Unless the true difference in treatment effects were much larger than expected, the study would be unlikely to end early.

To illustrate futility, when treatment B has the lower SLAM scores, cutoff Z-values to stop early and accept H0 (futility) are −0.120 at the first interim analysis and −0.013 at the second interim analysis. No futility value is needed for the final analysis.

This example illustrates some of the details that enter a simulation for a 1-year study design with an option to stop for futility. Using the R language we randomly generated 2000 data sets for each hypothesis. We assumed that the recalibrated SLAM score varied from 0 to 80 and has a standard deviation of 6. Under H0 (no difference) we might expect both groups A and B to have mean recalibrated SLAM scores of 14 and both would decline to mean scores of 10 after 1 year. Under HA (alternative) the superior treatment A would decline to 9, making the final mean difference 10 – 9 = 1. We also needed to specify the correlation between baseline and subsequent results and a realistic effect size. Under HA, a single simulation yielded z-scores of 1.97, 2.51, and 2.09 at months 6, 9, and 12 that have associated *P*-values of 0.048, 0.012, and 0.037. Recall that the cutoff Z-values to reject H0 are 3.47, 2.45, and 2.00 with corresponding type I errors of 0.0005, 0.014, and 0.045. Then, under HA for this scenario, the study would correctly reject H0 at the second interim analysis, since the z-score 2.51 > cutoff 2.45. These observed z-scores would not have stopped the study for futility. Repeating the simulation 2000 times under H0 provides an approximation of type I error, the proportion of times we stop and reject H0. Doing the same under HA provides an estimate of power, the proportion of times we stop and reject H0.

## Discussion

The definition, a priori, of what constitutes a clinically important improvement and worsening disease activity by the ACR committee [18] is a milestone in the development of more efficient and safer trials in SLE. Methods such as uniform-width intervals can make an ordinal measure of SLE disease activity more like an interval scale suitable for group sequential trials. Several uniform-width alternatives should be examined. When this seems too arduous, then coarsening the ordinal outcome into a binary outcome gives up some information, but opens up group sequential designs.

The advances in computing have made available a vast array of possible study designs that can only be compared using extensive simulations. The highly flexible Bayesian designs also require information about observed distributions from previous trials. The O’Brien-Fleming designs can be implemented without simulation using published tables [5] and relatively few new concepts.

The US Food and Drug Administration (FDA) has taken a conservative approach to randomized clinical trials focusing on type I error. When FDA approval is not an issue, other criteria may matter more. For example, a hospital may wish to save money by using the least expensive of medications that appear almost equally effective. The decision might give great weight to potential side effects or to finding the subgroups of patients who best tolerate each medication.

Sequential designs are a type of adaptive design. Adaptive designs deal with issues that may arise during a trial, such as poor recruitment, serious protocol violations, and unanticipated rates of adverse events [8]. Adaptive designs require pre-specified options, such as plans to modify dosage, drop study arms, change the random allocation, and change eligibility criteria during the trial.

In conclusion, group sequential randomized clinical trials may save time and resources. Modifying ordinal outcome scales for SLE, such as SLAM, BILAG, and SLEDAI, to give them interval properties, could facilitate the adoption of such study designs for comparing treatments for SLE.

## Note

This article is part of the series ‘*Measuring meaningful change in lupus clinical trials’*, edited by Matthew Liang and Chan-Bum Choi. Other articles in this series can be found at http://arthritis-research.com/series/trials

## Abbreviations

ACR: American College of Rheumatology
BILAG: British Isles Lupus Assessment Group
FDA: Food and Drug Administration
H0: null hypothesis
HA: alternative hypothesis
SLAM: Systemic Lupus Activity Measure
SLE: systemic lupus erythematosus
SLEDAI: Systemic Lupus Erythematosus Disease Activity Index
